# Editorial: Pharmacological Treatments Affecting Gastro-Intestinal Motility in Man

**DOI:** 10.3389/fphar.2021.801136

**Published:** 2022-01-19

**Authors:** E. Scarpellini, G. Ianiro, J. Tack

**Affiliations:** ^1^ Translational Research Center for Gastrointestinal Diseases (TARGID), University of Leuven, Leuven, Belgium; ^2^ Internal Medicine and Gastroenterology Unit, Gemelli University Hospital, Rome, Italy

**Keywords:** esophageal motility, gastrointestinal motility, diarrhea, constipation, gut microbiota

Motility of the upper and lower gastrointestinal (GI) tract is a complex of processes regulated by several receptors detectable along the GI tract e.g., cannabinoid, opioid, TRPV1 (Janssen et al., 2011), nerve endings and receptors belonging to the enteric nervous system (ENS), and hormones such as motilin, ghrelin, cholecystokinin (Janssen et al., 2011; Tack et al., 2016).

The upper and lower GI tract move in a highly coordinated fashion with periods of silence and periods of increased or maximum activity. This has several physiological functions: food boluses can progress from the esophagus to the anus. On the other hand, transient lower oesophageal sphincter relaxations guarantee the maintenance of the gastro-esophageal barrier towards acid and/or non-acid reflux of gastric/duodenal content (Tack and Pandolfino, 2018). Gastric relaxation, gastric meal accommodation, and colonic relaxation allow storage of larger amounts of luminal content. In the upper gastrointestinal tract, phases of interdigestive motility complex participate in the regulation of hunger, return of hunger, and satiety and appetite (Carbone and Tack, 2014). Continuous phasic motility of the stomach, small bowel, and colon favor digestion and absorption of vitamins and nutrients (Janssen et al., 2011). Several neuronal and non-neuronal receptors determine GI sensitivity to intestinal content, food ingredients, and, last but not least, gut microbiota and its products (Ianiro et al., 2014).

The gut microbiota is a “microorganism” itself with the GI tract, including the buccal cavity. It is composed of bacteria, viruses, protozoa, archaea, fungi (Scarpellini et al., 2015). It has metabolic, immune-modulating, and absorptive/digestive functions and regulates the secretion of GI hormones, hunger, and appetite in obese and non-obese animal models. There is a close interaction between digestive, sensory, and propulsive processes in the gastrointestinal tract and the resident microflora (Gibiino et al., 2017). Derangements of gut microbiota composition have been implicated in the pathogenesis of functional GI disorders such as irritable bowel syndrome, FD, and chronic constipation (Tait and Sayuk, 2021). Thus, the gut microbiota is a notable therapeutic target for the treatment of upper and lower functional GI disorders (e.g., irritable bowel syndrome). Gut microbiota can be modulated via absorbable and non-absorbable antibiotics, and pre- and probiotics. The latter has been deeply investigated in the last 3 decades by the pharmacological industry (Ortigão et al., 2020).

Special attention is needed for potential developments of fecal microbiota transplantation (FMT). This treatment, now commonly used to treat *Clostridium difficile* infections, seems to pave the road to its usage in functional GI disorders too (e.g. IBS) (Cammarota et al., 2019).

IBS, FD, and GERD show impairments of GI motility at various extents (Black et al., 2020). GERD often benefits from PPI treatment. However, about one-third of patients results to be refractory to treatment. Several prokinetic and reflux inhibitor remedies have been ruled out with alternate and incomplete success (Black et al., 2020). FD treatment is as complex as its physiopathology and prokinetics, drugs acting on GI sensitivity and CNS have an incomplete response. IBS, diarrhea, constipation treatment accounts of pre-, probiotics, antibiotics, antisecretory, prokintecis drugs, awaiting a personalized flow-chart.

Thus, several grey zones continue to exist in the neuro-gastroenterology field, such as treatment of oesophageal motility disorders (e.g., ineffective oesophageal motility disorders (IEM), achalasia, diffuse esophageal spasm, etc.) and refractory GERD (Mittal and Vaezi, 2020), and treatment of gastric (e.g., FD, gastroparesis) and lower GI tract motility disorders (e.g., IBS, diarrhea, constipation) (Camilleri, 2020; Scott et al., 2020).

Several prokinetic and non-prokinetic remedies have been studied and developed for the modulation of upper and lower GI motility. Their impact on the grey zones of neuro-gastroenterology was shown to be significantly different and heterogeneous (Black et al., 2020). These findings are increasingly allowing a patient-specific therapeutic approach.

The present article collection was selected to address several of the grey zones in clinical GI motility modulation, by pharmacological as well as non-pharmacological treatments.

We hope that this special issue will be useful for different specialists in the field (biologists, bio-engineers, doctors). It provides an extensive and updated review of the literature on the evidence on pharmacological and non-pharmacological treatments affecting gastrointestinal motility in humans. This collection can also serve as a basis for all future researchers in this expanding and rapidly changing area of biomedical investigation.
